# Differential effects of luteolin and its glycosides on invasion and apoptosis in MDA-MB-231 triple-negative breast cancer cells

**DOI:** 10.17179/excli2019-1459

**Published:** 2019-09-02

**Authors:** Jiyon Lee, Su-Ho Park, Jintak Lee, Hyunwoo Chun, Myoung-Kwon Choi, Jae-Hwan Yoon, Thu-Huyen Pham, Ki Hong Kim, Taeho Kwon, Hyung-Won Ryu, Sei-Ryang Oh, Do-Young Yoon

**Affiliations:** 1 Department of Bioscience and Biotechnology, Konkuk University, 120, Neungdong-ro, Gwangjin-gu, Seoul 05029, Republic of Korea; 2 Natural Medicine Research Center, Korea Research Institute of Bioscience and Biotechnology, 30 Yeongudanji-ro, Ohsong, Cheongju 28116, Republic of Korea

**Keywords:** luteolin, breast cancer, tumor migration, invasion, apoptosis

## Abstract

Luteolin is known to have anticancer activity in various cancers. Recent studies have shown that luteolin glycosides, such as luteolin-8-*C*-β-fucopyranoside, 7-methoxy-luteolin-8-C-β-(6- deoxyxylopyranos-3-uloside) and luteolin-8-C-β-d-glucopyranoside, flavonoids that are present in *Arthraxon hispidus*, exert antimigratory and anti-invasive effects, but no cytotoxic effect in estrogen receptor-positive MCF7 breast cancer cells. In the present study, we further investigated and compared differential effects of luteolin and its glycosides in MDA-MB-231 triple-negative breast cancer cells. Luteolin suppressed the expression of matrix metalloproteinase-9 and inhibited migration and invasion in MDA-MB-231 cells treated with the tumor promotor 12-O-tetradecanoylphorbol-13-acetate at non-cytotoxic concentrations (0, 5, and 10 μM). Furthermore, at cytotoxic concentrations (20 and 40 μM), luteolin induced apoptosis via extrinsic and intrinsic pathways in MDA-MB-231 cells. However, luteolin glycosides did not exert any cytotoxic, antimigratory, or anti-invasive effect in MDA-MB-231 cells. In brief, luteolin had both antimetastatic and cytotoxic effects on MDA-MB-231 cells, whereas luteolin glycosides had no effect on this cell line. Taking together the present results and our previous findings on the differential effects of luteolin and its glycosides on MDA-MB-231 and MCF-7 breast cancer cells, luteolin and its glycosides can be suggested as a potential candidate for breast cancer therapy.

## Introduction

Breast cancer is one of the most common causes of cancer-related deaths, and the prevalence of breast cancer in women has been increasing worldwide (Jones et al., 2017[19]). Despite significant advances in early detection and diagnosis of breast cancer, the incidence of this disease remains high (Howell et al., 2014[17]). MDA-MB-231 cells, a well-known triple-negative breast cancer (TNBC) cell line, are classified as estrogen receptor (ER)-, progesterone receptor (PR)-, and human epidermal growth factor receptor 2 (HER2)-negative (Navratil et al., 2015[26]). TNBC has an increased likelihood of distant recurrence and is associated with higher mortality rates than non-TNBC (Goncalves et al., 2018[12]). As TNBC cells, MDA-MB-231 cells are highly aggressive, exhibit rapid tumor growth, and are highly metastatic.

Breast cancer cells detach themselves from primary tumors and invade adjacent healthy tissues (Choi et al., 2018[5]). To this end, cancer cells degrade the basement membrane, including the specialized extracellular matrix (ECM) (Insua-Rodriguez and Oskarsson, 2016[18]). ECM degradation is one of the hallmarks of tumor migration and invasion (He et al., 2016[16]). During this process, matrix metalloproteinases (MMPs) are secreted, which dismantle the ECM structure and facilitate cell migration and invasion (He et al., 2016[16]). Gelatinase MMP-9 is a crucial enzyme that induces migration and invasion in breast cancer cells (Radisky and Radisky, 2015[30]). MMP-9 can be activated by 12-O-tetradecanoylphorbol-13-acetate (TPA), a potent tumor promoter (He et al., 2014[15]). 

Apoptosis is a well-characterized process of programed cell death that can be induced through two main pathways, namely, extrinsic and intrinsic pathways (Savitskaya and Onishchenko, 2015[32]). The extrinsic pathway can be activated by death receptors, such as Fas, tumor necrosis factor receptor, and death receptor 5 (DR5), which bind to their ligands FasL, TNF, and TNF-related apoptosis-inducing ligand (TRAIL), respectively (Ashkenazi, 2015[2]). The binding between death receptors and their ligands leads to the recruitment of Fas-associated death domain and procaspase-8 (Sakamaki et al., 2015[31]). Procaspase-8 is rapidly activated via autocleavage, resulting in the activation of downstream effectors, such as caspase-3, which plays important roles in apoptosis (Shalini et al., 2015[34]). The intrinsic pathway, which is induced by cell stress, is related to mitochondrial potential (Deus et al., 2014[9]). The activation of this pathway is regulated by the balance between pro-apoptotic proteins (e.g., Bax, Bak) and anti-apoptotic proteins (e.g., Bcl-2 and Bcl-xL) in the mitochondria and cytosol and leads to the activation of caspase-9 and -3 (Adams, 2003[1]; Choi et al., 2012[6]). Cleaved caspase-3 inactivates poly(ADP-ribose) polymerase (PARP), which is involved in DNA damage repair (Shah et al., 2018[33]). Thus, inhibition of invasion and induction of apoptosis are significant anticancer processes in breast cancer cells.

Flavonoids are abundantly present in fruits, vegetables, and medicinal herbs, and comprise polyphenols with a diphenylpropane C6-C3-C6 structure (Takemura et al., 2013[35]). Furthermore, flavonoids contain two aromatic rings (A and B), each bearing one or more phenolic hydroxyl groups that are connected by a carbon bridge comprising three carbon atoms (Panche et al., 2016[27]). Luteolin, 3',4',5,7-tetrahydroxyflavone, is one of the major flavonoids and exerts various biological effects, including anti-allergic, anti-inflammatory, and anticancer effects, in breast cancer (Aziz et al., 2018[3]; Lin et al., 2008[24]). In addition, its derivatives, including luteolin-8-*C*-β-fucopyranoside (LU8C-FP), 7-methoxy-luteolin-8-C-β-(6-deoxyxylopyranos-3-uloside) (mLU8C-PU) and luteolin-8-C-β-d-glucopyranoside (orientin), which are *C*-glycosides of luteolin compounds with no *O*-glycoside, are well known to exert inhibitory effects on cell invasion in MCF-7 breast cancer cells (Kim et al., 2018[22][23]; Park et al., 2013[28]). In the present study, we investigated and compared the differential effects of luteolin and its glycosides on migration, invasion, and apoptosis in TNBC MDA-MB-231 cells. 

## Materials and Methods

### Reagents

Luteolin (> 97 % purity), orientin, phenazine methosulfate (PMS), and Hoechst 33342 staining solution were purchased from Sigma-Aldrich (St. Louis, MO). LU8C-FP and mLU8C-PU were purified and supplied by Dr. Sei-Ryang Oh (Korea Research Institute of Bioscience & Biotechnology, Ochang, Korea) as recently described (Kim et al., 2018[22]; Park et al., 2013[28]). CellTiter 96^®^ AQueous One Solution Cell Proliferation Assay (3-[4,5-dimethylthiazol-2-yl]-5-[3-carboxymethoxyphenyl]-2-[4-sulfophenyl]-2*H*-tetrazolium, MTS) reagent was obtained from Promega (Madison, WI). An Annexin V-FITC Apoptosis Detection Kit was purchased from BD Pharmingen (San Diego, CA). JC-1 (5,5',6,6'-tetrachloro-1,1',3,3'-tetraethyl benzimidazolyl carbocyanine iodide) was purchased from Enzo (Farmingdale, NY). A pan-caspase inhibitor (z-VAD-fmk; benzyloxycarbonyl-Val-Ala-dl-Asp-fluoromethylketone) was obtained from R&D Systems (Minneapolis, MN). Antibodies specific to PARP, caspase-3, caspase-8, caspase-9, Bax, Bcl-2, Bcl-xL, and horseradish-peroxidase (HRP)-conjugated anti-mouse IgG antibody were purchased from Cell Signaling Technology (Beverly, MA). Antibodies specific to GAPDH and *β*-actin and HRP-conjugated anti-goat IgG antibody were purchased from Santa Cruz Biotechnology (Santa Cruz, CA). HRP-conjugated anti-rabbit IgG antibody was obtained from AbClon (Seoul, South Korea). Enhanced chemiluminescence (ECL) solution was purchased from Amersham Bioscience (Buckinghamshire, UK). An Easy-BLUE Total RNA Extraction kit was purchased from iNtRon Biotechnology (Seoul, South Korea).

### Cell culture and luteolin treatment

The human breast cancer cell line MDA-MB-231 was purchased from the American Type Culture Collection (Manassas, VA). HaCaT normal keratinocytes were used as control cells. Cells were cultured in Dulbecco's modified Eagle's medium supplemented with heat-inactivated 10 % (v/v) fetal bovine serum (FBS), 100 U/mL penicillin, and 100 μg/mL streptomycin at 37 °C in a humidified atmosphere with 5 % CO_2_ and 95 % air. For luteolin treatment, luteolin stock solution (2.0 mg/mL in DMSO) was added to the culture medium at the indicated concentrations (the final DMSO concentration was less than 0.05 %) and cells were incubated in the medium for the indicated time periods.

### Cell viability assay

MDA-MB-231 and HaCaT cells were seeded at approximately 1.5 × 10^4^ cells per well in a 96-well plate and grown overnight. Then, the medium was replaced with medium containing various concentrations of luteolin and luteolin glycosides and the cells were cultured for another 24 h. The effect of luteolin and luteolin glycosides on cell viability was assessed using electron-coupling reagent containing MTS and PMS (99:1 ratio). These reagents were mixed with medium that was added to the plate at 100 μL per well, and the cells were incubated for an additional 1 h. The optical density at 492 nm was measured using a microplate reader (Apollo LB 9110; Berthold Technologies GmbH, Bad Wildbad, Germany). The percentage of viable cells was estimated relative to those in untreated controls. The cell viability assay was repeated thrice.

### Reverse transcription-polymerase chain reaction (RT-PCR)

MDA-MB-231 cells were seeded in a six-well plate at 2 × 10^5^ cells/well, incubated overnight, and then washed twice with PBS. The cells were pretreated with various concentrations of luteolin for 1 h and then treated or not with TPA (50 nM) for another 24 h. For RT-PCR, total RNA was isolated with an Easy-BLUE Total RNA Extraction kit. RNA (5 μg) was converted to cDNA using a ProSTAR^TM^ RT-PCR Kit (Stratagene, La Jolla, CA) and Oligo(dT) primers. For PCR, the following primers were used: MMP-9, 5'AATCTCACCGACAGGCAGCT-3' (sense) and 5'-CCAAACTGGATGACGTGTC-3' (antisense); Fas, 5'-AGGGATTGGAATTGAGGAAG-3' (sense) and 5'-ATGGGCTTTGTCTGTGTACT-3' (antisense); FasL, 5'-CAAGATTGACCCCGGAAGTA-3' (sense) and 5'-GGCCTGTGTCTCCTTGTGAT-3' (antisense); glyceraldehyde 3-phosphate dehydrogenase (GAPDH), 5'-TGATGACATCAAGAAGGTGGTGGAG-3' (sense) and 5'-TCCTTGGAGGCCATGTAGGCCAT-3' (antisense). GADPH was used as internal control.

### Matrigel invasion assay and migration assay

The upper chamber of a Transwell plate (8-mm pore size; Millipore, Billerica, MA) was coated with 100 μL of Matrigel (Sigma-Aldrich) at 37 °C for 15 min and then incubated at room temperature for 10 min. MDA-MB-231 cells (1.5 × 10^5^ cells/well) were seeded in the Matrigel-coated upper chamber for the Matrigel invasion assay and a non-coated upper chamber filled with serum-free medium for the migration assay. Cells were treated with luteolin for 1 h and then with TPA (50 nM) for 24 h. After incubation for 24 h, the serum-free medium was removed, and the cells on the upper side of the chamber were removed using cotton swabs. The cells that had migrated through the membrane were fixed and stained with a Diff-Quik Solution Kit (#38721; Sysmex, Tokyo, Japan). Images of the membrane were captured using a light microscope. Finally, 50 μL of 10 % acetic acid was added to each well, and the cells were incubated for approximately 1 min. The absorbance at 630 nm was measured using the Apollo LB 9110 microplate reader.

### Assessment of cell morphology

Cell morphology was observed under an inverted phase-contrast microscope. Apoptotic changes were detected by Hoechst staining. Cells were seeded on cover slips and treated with luteolin for 24 h. The cover slips were then washed with PBS, and the cells were fixed and permeabilized with 100 % acetone at room temperature for 10 min. After washing with PBS, the cells were stained with Hoechst staining solution at 37 °C for 20 min. The cover slips were then washed with PBS, dried completely, and mounted on microscope slides. The slides were then observed under a fluorescence microscope (BX61-32FDIC; Olympus, Tokyo, Japan).

### Flow-cytometric analysis 

Apoptosis was detected by flow cytometry using an Annexin V-FITC Apoptosis Detection Kit. Briefly, MDA-MB-231 cells were seeded in six-well plates at 2 × 10^5^ cells/well and incubated overnight to allow the cells to adhere. The cells were treated with various concentrations of luteolin for 24 h, harvested, and washed with PBS. After centrifugation, the pelleted cells were resuspended in Annexin Binding Buffer. The cells were double-stained with Annexin V-fluorescein isothiocyanate (FITC) and propidium iodide (PI) following the manufacturer's instructions. Early apoptosis was defined as annexin V+/PI- staining, and late apoptosis was defined as annexin V+/PI+ staining as determined by flow cytometry (FACSCalibur; Becton Dickinson & Co., Franklin Lakes, NJ) and CellQuest Pro software (BD Biosciences, San Jose, CA).

### Western blot analysis

MDA-MB-231 cells were seeded in a six-well plate at 2 × 10^5^ cells/well and incubated overnight. The cells were then washed twice with ice-cold PBS and scraped on ice. Whole cell lysates were prepared by incubation in lysis buffer [50 mM Tris (pH 7.4), 150 mM NaCl, 1 % NP40, 0.1 % SDS, 0.25 % SDC, 1 mM EDTA, 1 mM EGTA, 1 mM orthovanadate, aprotinin (10 μg/mL), and 0.4 mM phenylmethylsulphonyl fluoride] at 4 °C for 1 h. The lysates were centrifuged at 10,000 × *g *for 30 min at 4 °C, and the supernatants were collected. Equal amounts of proteins (50 μg) were subjected to 10 % sodium dodecyl sulfate polyacrylamide gel electrophoresis and transferred to polyvinylidene fluoride membranes. The membranes were incubated with blocking buffer [5 % non-fat dry milk in TBS containing 0.1 % Tween-20 (TBST)] at room temperature for 1 h. The membranes were then incubated with primary antibodies targeting PARP, caspase-3, caspase-8, caspase-9, Bax, Bcl-2, and Bcl-xL at 4 °C overnight and washed thrice with TBST for 10 min per wash. Then, the membranes were incubated with HRP-conjugated anti-rabbit or anti-mouse IgG secondary antibody at room temperature for 1 h. After washing thrice for 10 min in TBST, proteins were detected with ECL solution and X-ray film.

### Analysis of the mitochondrial transmembrane potential (MTP) 

The mitochondrial transmembrane potential (∆*Ψ*m) was measured by JC-1 staining and flow cytometry. JC-1 is a lipophilic cationic dye that can enter and accumulate in the mitochondrial matrix in healthy mitochondria, which are negatively charged. On the other hand, JC-1 cannot enter the mitochondrial matrix in apoptotic cells because the MTP is collapsed in these cells. Therefore, JC-1 does not aggregate in apoptotic cells and remains as monomers instead. The aggregated form of JC-1 emits orange fluorescence at 590 nm (FL-2), whereas the monomers emit green fluorescence at 525 nm (FL-1). MDA-MB-231 cells were seeded in six-well plates at 5 × 10^5^ cells/well and treated with various concentrations of luteolin. After 24 h, the supernatants were transferred to Eppendorf tubes, washed with warm PBS, and trypsinized. The cells were collected from the supernatants in the tubes. JC-1 (5 μg/ml) was added to the cells and mixed until the precipitate was completely dissolved and no longer visible. The cells were incubated in the dark at 37 °C for 10 min, centrifuged (300 ⨯ *g*, 5 min, 4 °C), washed twice with cold PBS, and resuspended in 200 μL of PBS. The cell solutions were sorted using a FACSCalibur instrument and data were analyzed using the CellQuest software. The entire protocol was carried out in reduced light conditions.

### Statistical analyses

All experiments were performed at least thrice. Data are presented as the mean ± standard deviations (SD). Data were analyzed by Student's *t*-test or one-way ANOVA followed by Tukey's honestly significant difference (HSD) test. *P* < 0.05 was considered statistically significant.

## Results

Luteolin is cytotoxic at high concentrations, but its glycosides do not exert cytotoxicity in MDA-MB-231 breast cancer cells. 

**Figure 1:**
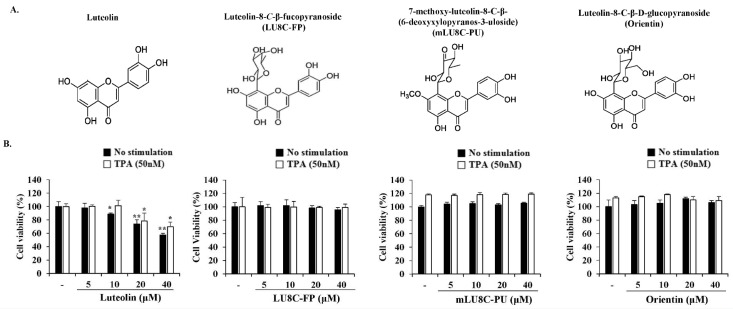
Effects of luteolin and its glycosides on the viability of MDA-MB-231 breast cancer cells. (A) Structures of luteolin, luteolin-8-*C*-β-fucopyranoside (LU8C-FP), 7-methoxy-luteolin-8-C-β-(6-deoxyxylopyranos-3-uloside) (mLU8C-PU), and luteolin-8-C-β-d-glucopyrano side (orientin). (B) MDA-MB-231 breast cancer cells were treated with luteolin or its glycosides in the presence or absence of TPA (50 nM) for 24 h. Cell viability was determined by MTS assay. Statistically significant differences between luteolin-treated versus non-treated cells were determined by two-tailed Student's *t*-test. *p < 0.05, **p < 0.005 (n = 3)

To investigate the cytotoxicity of luteolin and its glycosides, MDA-MB-231 breast cancer cells were treated with various concentrations of luteolin, LU8C-FP, mLU8C-PU, and orientin in the presence or absence of TPA (50 nM) for 24 h. Cell viability was then measured by MTS assay (Figure 1B(Fig. 1)). Luteolin exerted a cytotoxic effect at high concentrations (20 and 40 μM), but not at low concentrations (5 and 10 μM) in MDA-MB-231 cells. Its derivatives exhibited no cytotoxicity in MDA-MB-231 cells at all concentrations tested.

**Figure 2:**
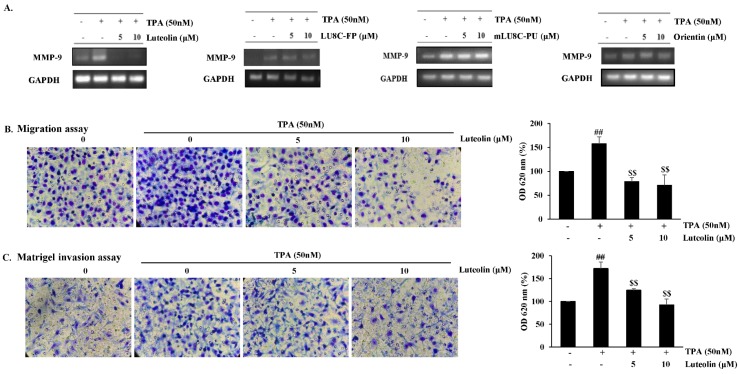
Luteolin inhibits migration and invasion in MDA-MB-231 breast cancer cells. MDA-MB-231 cells were pretreated with luteolin, LU8C-FP, mLU8C-PU, orientin, or TPA (50 nM) for 24 h. (A) MMP-9 mRNA expression levels were evaluated by RT-PCR. (B) Migration assay and (C) Matrigel invasion assay of TPA-stimulated MDA-MB-231 cells exposed to luteolin for 24 h. Statistical significance was analyzed by one-way ANOVA followed by Tukey's HSD test. *^##^p<0.005* (non-treated vs. TPA alone) and *^$$^p < 0.005* (TPA alone vs. TPA plus luteolin) (n = 3)

Luteolin, but not its glycosides, suppresses TPA-induced MMP-9 expression and inhibits migration and invasion in MDA-MB-231 breast cancer cells. MMP-9 is an important ECM-degrading enzyme and mediates cell migration and invasion (Yadav et al., 2014[36]). mRNA expression levels of TPA-induced MMP-9 were evaluated in MDA-MB-231 cells treated with luteolin at non-cytotoxic concentrations (0, 5, and 10 μM) or its glycosides for 24 h. Luteolin suppressed MMP-9 mRNA expression (Figure 2A(Fig. 2)) and inhibited migration (Figure 2B(Fig. 2)) and invasion (Figure 2C(Fig. 2)) in a dose-dependent manner in TPA-stimulated MDA-MB-231 cells. Its glycosides did not affect TPA-induced MMP-9 mRNA expression levels at various concentrations (0-40 μM) in MDA-MB-231 cells (Figure 2A(Fig. 2), data not shown for 20-40 μM). 

### Luteolin causes cell death by inducing the formation of apoptotic bodies in MDA-MB-231 cells

**Figure 3:**
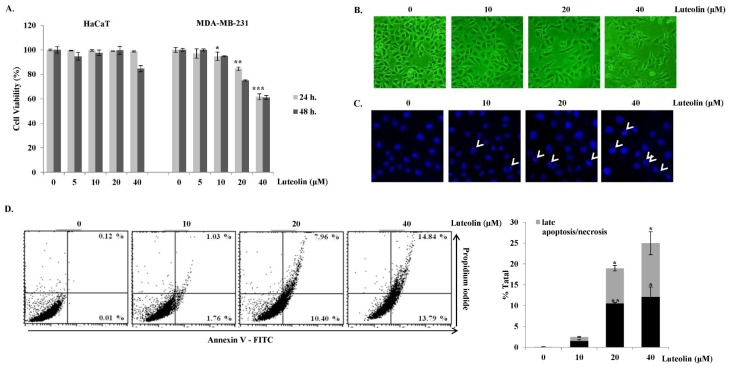
Cytotoxic and apoptotic effects of luteolin in MDA-MB-231 breast cancer cells. (A) MDA-MB-231 breast cancer cells and HaCaT normal keratinocytes were treated with luteolin for 24 or 48 h. Cell viability was determined by MTS assay. (B) Morphological changes in MDA-MB-231 cells after treatment with various concentrations (0, 10, 20, and 40 μM) of luteolin for 24 h were observed under a phase-contrast microscope (100×). (C) Apoptotic nuclei after treatment with luteolin were observed under a fluorescence microscope after Hoechst staining (100×). (D) Flow-cytometric analysis after staining with Annexin V-fluorescein isothiocyanate (FITC) and propidium iodide (PI). Percentages of significant events in early apoptosis (bottom right quadrants) and late apoptosis (top right quadrants). Bar graph represents annexin V+/PI- (early apoptotic) and annexin V+/PI+ (late apoptotic) cells (n = 3). Statistically significant differences between luteolin-treated versus non-treated cells were analyzed by two-tailed Student's *t*-test. *p < 0.05, **p < 0.005, ***p < 0.001 (n = 3)

As only luteolin exhibited cytotoxicity in MDA-MB-231 cells, we next focused on the apoptotic effect of luteolin in these cells. First, the cytotoxic effect of luteolin on MDA-MB-231 cells was verified by MTS assays after exposing the cells to various concentrations of luteolin (Figure 3A(Fig. 3)) for 24 and 48 h. Luteolin significantly decreased cell viability in a dose- and time-dependent manner in MDA-MB-231 cells, whereas it showed no significant cytotoxic effect on HaCaT human normal keratinocytes, demonstrating that luteolin exerts cytotoxic effects specifically in MDA-MB-231 cells. Further, it was verified that high concentrations of luteolin (20 and 40 μM) reduced cell viability in MDA-MB-231 cells within 24 h. Apoptosis is accompanied by cell shrinkage, DNA fragmentation, chromatin condensation, and the formation of apoptotic bodies, which are phagocytosed by macrophages (Zhivotosky and Orrenius, 2001[39]). Nuclear shrinkage can be detected by Hoechst staining, which binds to the minor grooves in DNA (Bak et al., 2013[4]). Inverted phase-contrast microscopy revealed morphological changes as well as growth inhibition upon luteolin treatment (Figure 3B(Fig. 3)). MDA-MB-231 cells were stained with Hoechst 33342 dye to detect apoptotic nuclei after luteolin treatment. Cells in the control group had round shapes and a blue color. However, luteolin-treated breast cancer cells were more condensed than the control cells (Figure 3C(Fig. 3), condensed cells are indicated by white arrows). Flow cytometry after Annexin V-FITC/PI double staining was used to confirm apoptosis. Luteolin induced early apoptosis and late apoptosis as indicated by annexin V+/PI- staining and annexin V+/PI+, respectively, in MDA-MB-231 breast cancer cells (Figure 3D(Fig. 3)). The above findings indicated that luteolin induces apoptosis by altering cell morphology and inducing apoptotic body formation in MDA-MB-231 breast cancer cells.

### Luteolin induces apoptosis through the caspase cascade and PARP inactivation in MDA-MB-231 breast cancer cells

**Figure 4:**
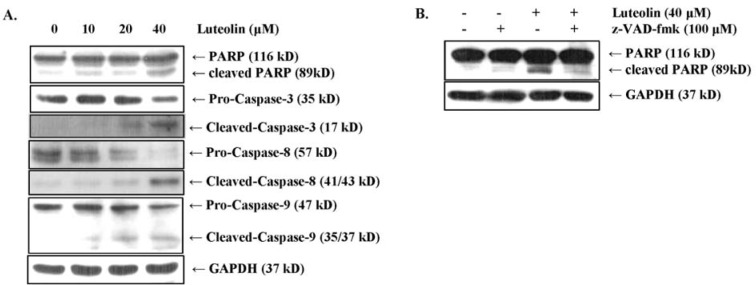
Luteolin induces apoptosis via the caspase cascade and PARP degradation in MDA-MB-231 breast cancer cells. (A) Processing of caspases-8, -9, and -3 and PARP were detected by Western blot analysis. (B) MDA-MB-231 breast cancer cells were pretreated with 100 μM z-VAD-fmk (pan-caspase inhibitor) for 2 h and subsequently stimulated with 40 μM luteolin. PARP levels were analyzed by Western blot analysis, and GAPDH was used as an internal control.

The caspase cascade is a crucial signaling pathway that mediates apoptotic cell death through proteolytic processing (Duclos et al., 2017[10]). Therefore, to analyze whether luteolin induces caspase-dependent PARP inactivation, we measured the protein levels of caspases and PARP by Western blot analysis. Luteolin treatment led to reduced protein levels of the precursor forms of caspase-8, -9, and -3, whereas the protein levels of their cleaved forms were increased (Figure 4A(Fig. 4)). In addition, cleaved PARP levels were increased in luteolin-treated compared to non-treated MDA-MB-231 cells (Figure 4A(Fig. 4)). Western blot analysis of cells pretreated with the pan-caspase inhibitor z-VAD-fmk (100 μM) confirmed that proteolytic (40 μM) luteolin-induced PARP cleavage was inhibited by z-VAD-fmk (Figure 4B(Fig. 4)). The above findings demonstrated that luteolin suppresses the growth of MDA-MB-231 cells through the induction of apoptosis via PARP inactivation in a caspase-dependent manner. 

### Luteolin promotes the extrinsic and intrinsic apoptotic pathways in MDA-MB-231 breast cancer cells

**Figure 5:**
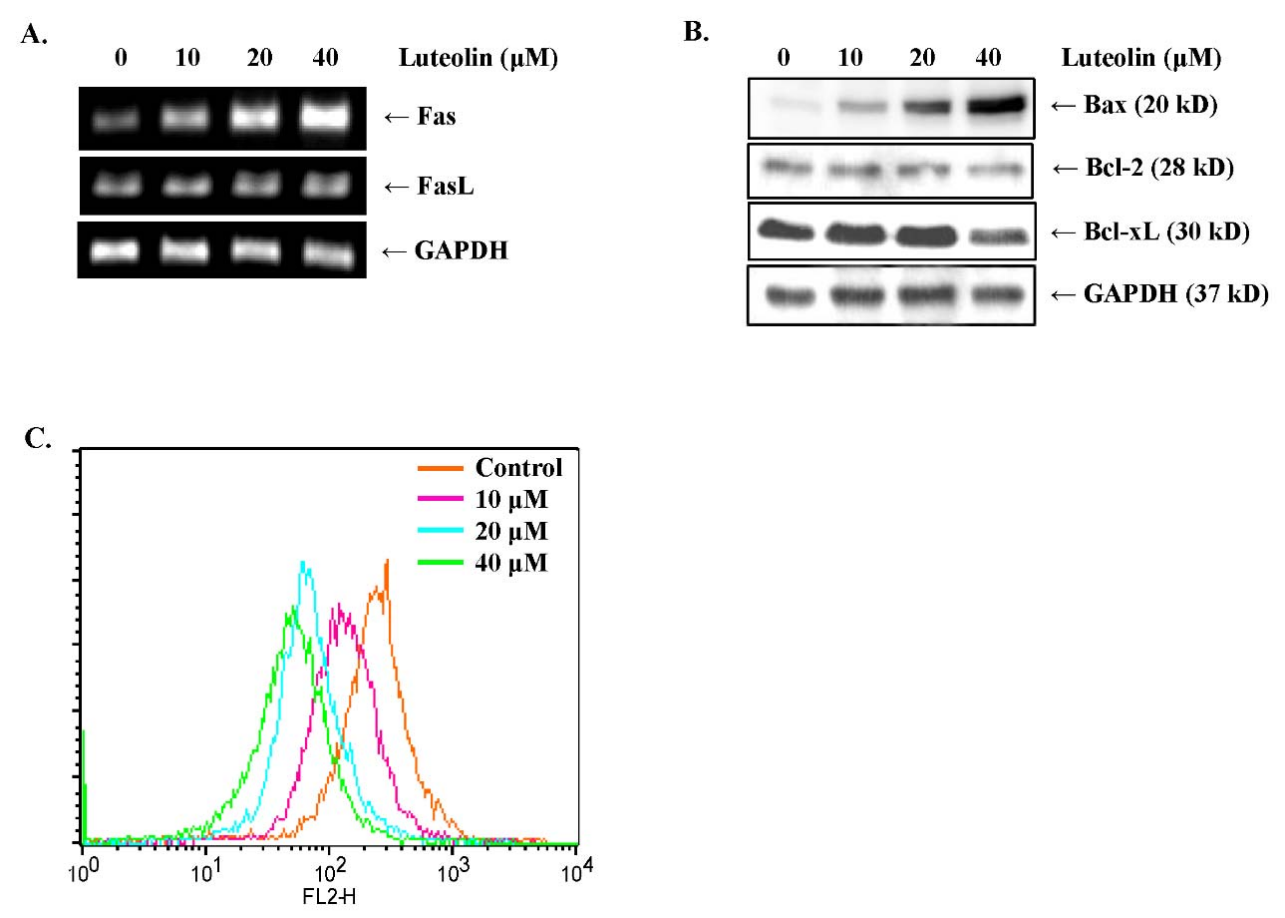
Luteolin induces apoptosis via the extrinsic and intrinsic pathways in MDA-MB-231 breast cancer cells. (A) mRNA expression levels of Fas death receptor and Fas ligand (FasL) were analyzed by RT-PCR. (B) Protein expression levels of Bax, Bcl-2, and Bcl-xL were detected by Western blot analysis. (C) The mitochondrial transmembrane potential (∆*Ψ*m) was analyzed by JC-1 staining after treating the cells with luteolin for 24 h. The histogram shows the distribution of JC-1. Orange color (FL2-H, right) represents JC-1 aggregates, which are a characteristic feature of healthy cells; green color (FL2-H, left) represents JC-1 monomers, which are a characteristic feature of apoptotic cells.

Cell apoptotic pathways can be classified into the death receptor pathway (extrinsic) and the cell stress pathway (intrinsic) (Derakhshan et al., 2017[8]). The extrinsic apoptotic pathway is associated with death receptor signaling (Green and Llambi, 2015[13]). To elucidate the mechanisms by which luteolin activates the extrinsic pathway of apoptosis, we measured the mRNA levels of representative death receptors, including Fas and DR5, by RT-PCR. In MDA-MB-231 breast cancer cells, luteolin treatment upregulated Fas mRNA expression in a dose-dependent manner (Figure 5A(Fig. 5)); however, DR5 mRNA levels were not affected (data not shown). In addition, FasL expression was slightly upregulated in MDA-MB-231 cells treated with luteolin (Figure 5A(Fig. 5)). The intrinsic pathway is regulated by the Bcl-2 family of proteins, which are located near the mitochondrial membrane (Edlich, 2018[11]). Bax increases mitochondrial membrane permeability by forming oligomers at the outer mitochondrial membrane to induce apoptosis (Pena-Blanco and Garcia-Saez, 2018[29]). As shown in Figure 5B(Fig. 5), Western blot analysis revealed that Bax expression was increased, whereas Bcl-xL expression was downregulated by luteolin in MDA-MB-231 cells. To validate the Western blotting results, we investigated mitochondrial transmembrane potential (∆*Ψ*m) using JC-1 dye. In healthy cells with a normal ∆*Ψ*m, JC-1 aggregates in the mitochondria and emits red fluorescence, whereas in apoptotic cells, JC-1 monomers leak to the cytoplasm because of the low ∆*Ψ*m and emit green fluorescence (Marcondes et al., 2019[25]). Luteolin treatment decreased the ∆*Ψ*m in MDA-MB-231 cells as evidenced by the fact that fluorescence signals gradually shifted to green (Figure 5C(Fig. 5)). The above results indicated that luteolin induces apoptosis via the Fas-mediated extrinsic pathway and the mitochondrial-mediated intrinsic pathway in MDA-MB-231 breast cancer cells.

## Discussion

Luteolin and its glycosides, including LU8C-FP, mLU8C-PU, and orientin, have been reported as potential anticancer compounds in several breast cancer cell lines, including wild-type ER (+) MCF-7 cells (Kim et al., 2012[21], 2018[22][23]; Park et al., 2013[28]). O-glycosides reportedly are well hydrolyzed by intestinal microfloral *Lactococcus* sp. and *Enterococcus* sp., whereas flavone C-glycosides are not easily hydrolyzed by the intestinal microflora (Kim et al., 2015[20]). 8-C flavones might be absorbed in the small intestine and transported to metastatic regions of ER (+) breast cancer, where these 8-C flavones can suppress MMP-9 and IL-8 (Kim et al., 2012[21], 2018[22][23]; Park et al., 2013[28]). In this study, we evaluated and compared the effects of luteolin and its glycosides (8-C flavones) in the MDA-MB-231 TNBC cells, which are more aggressive than the ER (+) MCF-7 breast cancer cells. TPA, a well-known tumor promoter (He et al., 2014[15]), promotes breast cancer metastasis by activating various intracellular factors, including MMP-9 (Park et al., 2013[28]). Therefore, the TPA-treated MDA-MB-231 cell model was used in this study to investigate the effects of the above compounds.

This study revealed that luteolin at high concentrations (20 and 40 μM) had a cytotoxic effect in MDA-MB-231 cells, whereas its glycosides had no cytotoxic effect at all concentrations tested. Furthermore, luteolin suppressed migration and invasion, likely by downregulating MMP-9 expression at non-cytotoxic concentrations (5 and 10 μM), which in turn might have inhibited ECM degradation in MDA-MB-231 cells. However, luteolin glycosides did not affect migration and invasion at the same concentrations in MDA-MB-231 cells. Collectively, these results showed that only luteolin, not its glycosides, inhibited migration and invasion in MDA-MB-231 cells. While luteolin glycosides target the ER-mediated signaling pathway in ER (+) MCF-7 cells, these compounds might not inhibit migration and invasion signaling pathways mediated via MMP-9 in MDA-MB-231 TNBC breast cancer cells. In a previous study, a relatively low concentration (10 μM) of luteolin significantly inhibited vascular endothelial growth factor secretion in MDA-MB-231 cells (Cook et al., 2017[7]). It has been also reported that luteolin is rapidly absorbed after administration and may circulate in humans (Yasuda et al., 2015[37]), supporting the hypothesis that luteolin might be transported to TNBC metastatic regions, where it might exert inhibitory effects on migration and invasion as observed in our *in vitro* experiments.

Next, we investigated the effects of luteolin-induced cell death in MDA-MB-231 breast cancer cells. At high concentrations of luteolin, cell growth was inhibited, cells became more rounded, and apoptotic nuclei were detected in MDA-MB-231 cells. Moreover, luteolin significantly induced early and late apoptosis in a dose-dependent manner. Apoptosis can be induced either by activation of death receptors or by disturbance of mitochondria through the activation of caspases (Hassan et al., 2014[14]). In this study, we evaluated whether luteolin induced any of these apoptotic pathways in MDA-MB-231. Luteolin treatment led to the degradation of caspases in MDA-MB-231 breast cancer cells. Luteolin-induced PARP cleavage was inhibited when MDA-MB-231 cells were treated with a pan-caspase inhibitor. These results demonstrate that luteolin induces apoptosis by caspase-dependent inactivation of the DNA repair protein PARP.

Extrinsic apoptosis can be initiated upon activation of death receptors or ligands, such as Fas/FasL (Green and Llambi, 2015[13]). FasL expression was slightly increased, Fas expression was significantly enhanced, and procaspase-8 was cleaved in luteolin-treated MDA-MB-231 cells. The intrinsic apoptotic pathway involves an increase in mitochondrial membrane permeability, downregulation of anti-apoptotic proteins, upregulation of pro-apoptotic proteins (Zhang et al., 2017[38]), and cytochrome c release, followed by the activation of caspase-9 and consequently, activation of caspase-3 (Green and Llambi, 2015[13]). Luteolin induced the intrinsic apoptotic pathway, likely by increasing Bax expression and decreasing Bcl-2 and Bcl-xL expression in MDA-MB-231 cells. Further, procaspase-9 was cleaved to the active form, caspase-9, and the ∆*Ψ*m was reduced in luteolin-treated MDA-MB-231 cells. Thus, luteolin might have induced the formation of Bax oligomers at the outer mitochondrial membrane and thus increased the mitochondrial membrane permeability, resulting in apoptosis.

## Conclusions

In summary, we investigated and compared the differential antimigratory, anti-invasive and cytotoxic effects of luteolin and its glycosides on breast cancer cells. Luteolin inhibited tumor migration and invasion by suppressing MMP-9 in MDA-MB-231 cells at non-cytotoxic concentrations. Cytotoxic concentrations of luteolin induced apoptosis via the Fas/FasL-mediated extrinsic and mitochondria-related intrinsic pathways in MDA-MB-231 cells. However, luteolin glycosides were not cytotoxic in MDA-MB-231 cells. Therefore, luteolin and its glycosides have potential as therapeutic agents for breast cancer treatment.

## Notes

Jiyon Lee and Su-Ho Park contributed equally to this article.

## Acknowledgements

This research was supported by Konkuk University in 2019.

## Conflict of interest

The authors declare that they have no conflict of interest. 

## Ethical approval

This article does not contain any studies with human participants or animals performed by any of the authors.
